# Efficacy of steroid therapy for Fukuyama congenital muscular dystrophy

**DOI:** 10.1038/s41598-021-03781-z

**Published:** 2021-12-20

**Authors:** Terumi Murakami, Takatoshi Sato, Michiru Adachi, Kumiko Ishiguro, Minobu Shichiji, Hisateru Tachimori, Satoru Nagata, Keiko Ishigaki

**Affiliations:** 1 Department of Pediatrics, Tokyo Women’s Medical University School of Medicine, 8-1 Kawada-cho, Shinjuku, Tokyo 162-8666 Japan; 2 Department of Clinical Research, National Hospital Organization Higashisaitama National Hospital, 4147 Kurohama, Hasuda, Saitama 349-0196 Japan; 3 Department of Rehabilitation, Tokyo Women’s Medical University, 8-1 Kawada-cho, Shinjuku, Tokyo 162-8666 Japan; 4 Department of Clinical Epidemiology, Translational Medical Center, National Center of Neurology and Psychiatry, 4-1-1 Ogawa-Higashi, Kodaira, Tokyo 187-8551 Japan

**Keywords:** Drug discovery, Medical research, Neurology

## Abstract

Although there is only symptomatic treatment for Fukuyama congenital muscular dystrophy (FCMD), several reports have suggested that steroid therapy could be effective for FCMD; however, no independent intervention studies have been conducted. This study aimed to evaluate the efficacy of steroid therapy for restoring motor functions in FCMD patients. This study involved 3-to-10-year-old FCMD patients who exhibited a decline in motor functions, requested steroid therapy. Patients with consent started oral administration of 0.5-mg/kg prednisolone every alternate day, which was increased to 1.0 mg/kg if the response was inadequate. We used the Gross Motor Function Measure (GMFM) to evaluate and compare the motor functions of all patients. Wilcoxon signed-rank test (significance level, *P* ≤ 0.05) was used for statistical analysis. At the onset of steroid therapy, 8.10 years (SD, 2.14 years) was the mean age of FCMD patients. The mean GMFM difference between before and after the steroid therapy was + 1.23 (SD, 1.10), and a *P* value of 0.015 represented significant improvement in GMFM. Our results indicate that steroid therapy may contribute to the maintenance and improvement of the motor functions of advanced-stage FCMD patients.

**Clinical Trial Registration** Registration Number: UMIN000020715, Registration Date: Feb 1st, 2016 (01/02/2016).

## Introduction

Fukuyama congenital muscular dystrophy (FCMD) is the second most frequent type of childhood muscular dystrophy in Japan, after Duchenne muscular dystrophy (DMD)^1,2^. This disease is caused by mutations in the fukutin (*FKTN*) gene. The fukutin protein acts as a transferase for ribitol phosphate^3^. A defect in fukutin induces abnormalities in the *O*-mannose-type sugar chain of α-dystroglycan (α-DG)^4^. This abnormality reduces the binding to laminin within the basal membrane, thus resulting in the weakening of myocyte membranes and necrosis/denaturation of muscle cells. Overall, 87% of patients with FCMD exhibit a so-called founder mutation in *FKTN* (a 3-kb homozygous insertion mutation in the 3′ untranslated region). In the remaining patients, heterozygous mutations (a combination of a founder mutation and a point mutation) are observed^5^.

Patients with FCMD exhibit generalized muscle weakness and psychomotor retardation from early infancy^2,6^. In these patients, maximal motor ability is observed at ages 2–8, with 15% of the patients acquiring the ability to walk at the peak point (mild form). Moreover, 75% of the patients are only able to sit on their own or do bottom shuffling (typical form), and 10% of the patients lack head control (severe form)^7,8^. After the peak point, motor functions start regressing and never recover spontaneously. After reaching the age of 15, many patients become bedridden^7^. This is an intractable disease with no established cure, and patients often die of respiratory failure or cardiomyopathy before age 20. In recent years, the functions of the *FKTN* gene product have been clarified, and studies of genetic therapy involving α-dystroglycan-related treatment strategies and radical treatment have been ongoing^3,4^. Nevertheless, these findings have not resulted in practical applications, and no therapeutic methods are available for suppressing the progression of muscle weakness or other symptoms.

In patients with DMD, the stability of the muscular basal membrane and cellular membrane is disrupted because of the absence of dystrophin, which results in progressive muscle fiber damage. The efficacy of steroid therapy in managing this disease has been demonstrated, as it extends the walking duration, maintains the cardiopulmonary functions, and decreases the incidence of scoliosis^9–12^. The possible mechanisms to explain the efficacy of steroid therapy include decrease in cytotoxic T cells, Ca intracellular influx, and laminin protein concentrations; however, these mechanisms remain unclear^10^.

FCMD is also caused by a disruption in the stability of the basement membrane and cell membrane because of abnormalities in the sugar chain of α-dystroglycan. The potential efficacy of steroid therapy for FCMD has been suggested in several case reports^13–17^. Moreover, it has been reported that short-term steroid administration is also effective in improving the exacerbated muscle weakness after viral infection in patients with FCMD^13–17^. In this study, we planned a prospective study to evaluate the efficacy of oral steroid therapy in patients with FCMD.

## Results

### Patients

This study involved nine patients (four boys and five girls), eight of whom had a 3-kb homozygous insertion mutation in the *FKTN* gene, whereas the remaining patient had a composite heterozygous mutation (Table 1). One patient was able to walk at the peak motor condition (mild form), seven patients attained the ability to sit unaided and for bottom shuffling (typical form), and one patient had not achieved head control (severe form) (Table 1). The findings of declined motor functions included three cases of deteriorating lower limb movement, five cases of deteriorating arm movement, six cases of unsteady trunk, four cases of unsteady head control, and one case of walking difficulty (Table 2).

**Table 1:** Patient characteristics.

Patient no	Sex	*Fukutin* gene 3 kb insertion mutation	Clinical form	Peak motor performance
1	F	Homozygosity	Mild	Walking
2	F	Homozygosity	Typical	Bottom shuffling
3	M	Homozygosity	Typical	Bottom shuffling
4	F	Homozygosity	Typical	Bottom shuffling
5	M	Homozygosity	Typical	Bottom shuffling
6	F	Homozygosity	Typical	Bottom shuffling
7	M	Homozygosity	Typical	Bottom shuffling
8	F	Homozygosity	Typical	Bottom shuffling
9	M	Heterozygosity	Severe	Unsteady head control

*F* female, *M* male.

**Table 2:** Regression findings before steroid therapy.

Patient no.	Head control	Trunk stability	Foot movement	Upper limb movement	Able to sit	Able to turn over	Increased speed of bottom shuffling	Able to crawl on all fours	Able to walk longer distance
1	Stable	**Worsened**	Stable	Stable	Stable	Stable	Stable	**Worsened**	**Worsened**
2	**Worsened**	Stable	**Worsened**	Stable	Stable	Stable	Stable	N.A.	N.A.
3	**Worsened**	**Worsened**	Stable	**Worsened**	Stable	Stable	Stable	N.A.	N.A.
4	Stable	**Worsened**	Stable	Stable	Stable	**Worsened**	Stable	N.A.	N.A.
5	**Worsened**	Stable	**Worsened**	**Worsened**	Stable	**Worsened**	Stable	N.A.	N.A.
6	Stable	Stable	**Worsened**	**Worsened**	Stable	Stable	Stable	N.A.	N.A.
7	**Worsened**	**Worsened**	Stable	Stable	Stable	N.A	N.A	N.A.	N.A.
8	Stable	**Worsened**	Stable	**Worsened**	Stable	Stable	Stable	N.A.	N.A.
9	Stable	**Worsened**	Stable	**Worsened**	N.A.	N.A.	N.A.	N.A.	N.A.

*N.A.* Not applicable.

Significant values are in [bold].

### Steroid therapy

The mean age at steroid therapy onset was 8.10 years (SD, 2.14 years) (Table 3). Prednisolone was administered to two patients at 0.5 mg/kg on an alternate-day basis, to one patient at 0.7 mg/kg on an alternate-day basis, and to six patients at 1.0 mg/kg on an alternate-day basis (Table 3). The mean duration of steroid administration was 8.86 months (SD, 3.05 years) (Table 3). One additional month was necessary to confirm the drug compliance, and the evaluation after therapy was delayed. Particularly, in case 6, the parents of the patient were very worried about her irritability and the increase in the dose of prednisolone was time consuming; moreover, the dose could only be increased to 0.7 mg/kg. Case 2 was evaluated in a short period because this study was terminated because of the enforcement of the Clinical Trials Act.

**Table 3:** Results of the steroid therapy.

Patient no.	Age at initiation of steroid therapy (years)	Prednisolone dose (mg/kg on an alternate-day basis)	Administration duration (months)
1	7.8	1.0	10.8
2	10.9	0.5	3.0
3	10.9	1.0	7.3
4	9.3	1.0	9.4
5	8.4	0.5	8.0
6	8.3	0.7	13.7
7	6.5	1.0	8.9
8	6.5	1.0	7.0
9	4.3	1.0	11.4
Average	8.10 (2.14 SD)		8.86 (3.05 SD)

*SD* standard deviation.

The adverse events that occurred during the steroid therapy included four cases of irritability/agrypnia, one case of increased appetite, and one case of moon-shaped face (Table 4). None of these adverse events were sufficiently serious to warrant the discontinuation/suspension of steroid therapy.

**Table 4:** Adverse events during steroid therapy.

Patient no.	Agrypnia	Increased appetite	Moon face	Irritability
1	**Applicable**	**Applicable**	**Applicable**	N.A.
2	N.A.	N.A.	N.A.	N.A.
3	**Applicable**	N.A.	N.A.	N.A.
4	N.A.	N.A.	N.A.	N.A.
5	N.A.	N.A.	N.A.	N.A.
6	N.A.	N.A.	N.A.	N.A.
7	N.A.	N.A.	N.A.	N.A.
8	N.A.	N.A.	N.A.	**Applicable**
9	**Applicable**	N.A.	N.A.	N.A.

*N.A.* Not applicable.

Significant values are in [bold].

### GMFM evaluation

**Figure 1:**
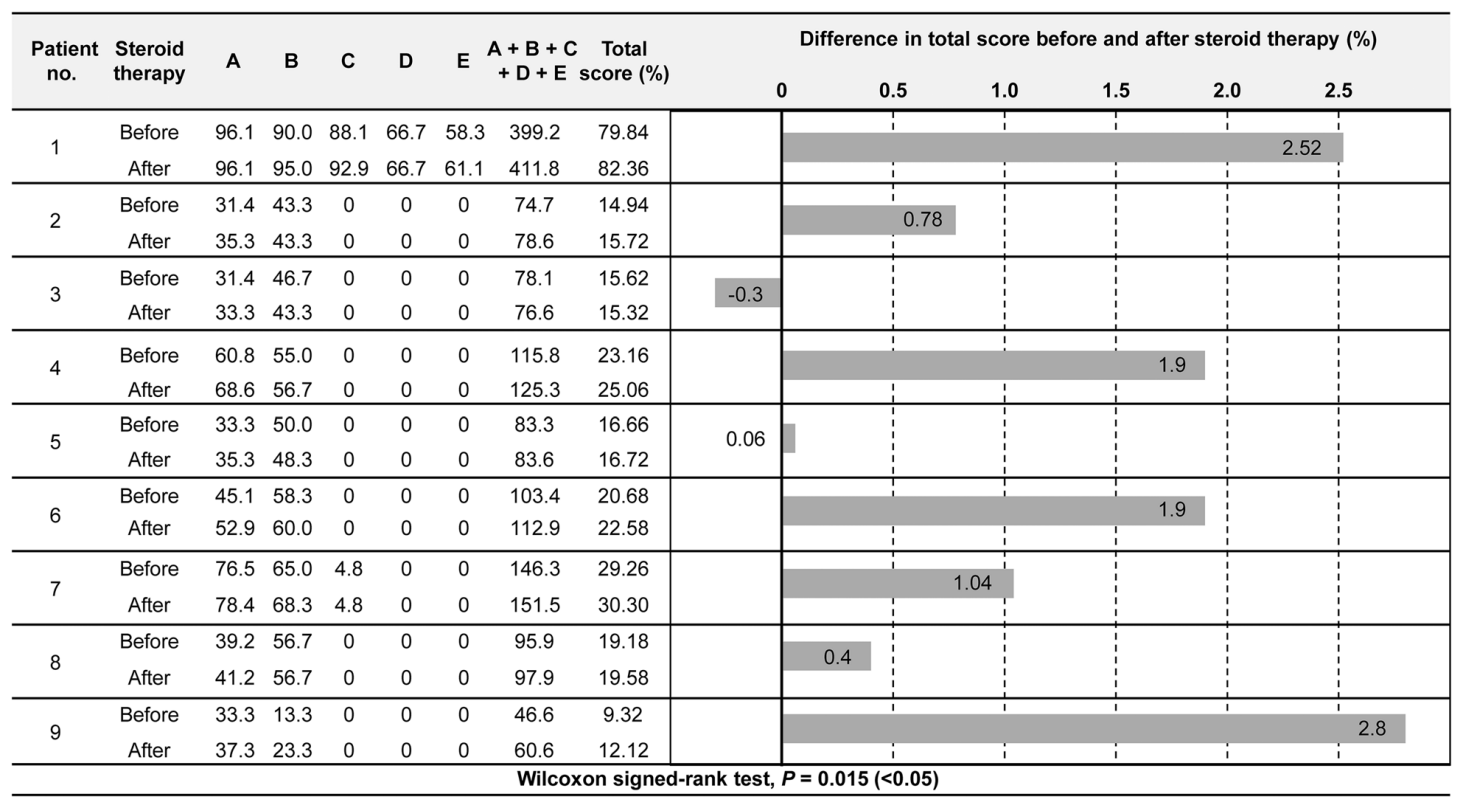
Comparison of GMFM results before and after steroid therapy.

For dimension A of the Gross Motor Function Measure (GMFM)^18^, eight of the nine cases showed increased percent scores, whereas one case remained unchanged (Fig. 1). For dimension B, five of the nine cases showed increased percent scores, two remained unchanged, and two exhibited decreased percent scores (Fig. 1). The two cases with decreased percent scores for dimension B exhibited increased scores for dimension A (Fig. 1). Of the two cases that could be evaluated for dimension C, one case had an increased percent score, and one remained unchanged (Fig. 1). The one case that could be evaluated for dimension D exhibited an increased percent score (Fig. 1). From before to after the steroid therapy, the mean change in the total score was + 1.23 (SD, 1.10), with eight of the nine cases exhibiting an increase in the total scores (Fig. 1). In case 3, the total score was decreased, but the percent score in dimension A was increased (Fig. 1). The Wilcoxon signed-rank test revealed significant differences in the motor functions between before and after the steroid therapy (*P* = 0.015; Fig. 1).

## Discussion

It is known that the motor functions of patients with FCMD peak from 2 to 8 years of age and start regressing at around 4–8 years of age. Once regression starts, it is understood that the motor functions never recover^1^. In the present study, we administered a steroid orally to nine patients with FCMD whose motor functions had started regressing and observed improvements in all of them. This result suggests that steroid therapy may help these patients improve or maintain their motor functions after they have already started regressing. While previous case studies focused on patients with mild FCMD, including cases that gained the ability to walk^13,14^, we administered steroid therapy not only to mild cases, but also to typical and severe forms. Consequently, we observed improved motor functions in patients of all clinical forms. In the mild and typical forms, the patients gained the ability to perform actions that were previously impossible (Table 5). In the severe cases, the patients were able to stabilize the previously unsteady neck and trunk and sit unaided, showing an improvement in the peak motor ability (Table 5).

**Table 5:** Improved and acquired findings after steroid therapy.

Patient no.	Steroid therapy	Head control	Trunk stability	Foot movement	Upper limb movement	Able to sit	Able to turn over	Increased speed of bottom shuffling	Able to crawl on all fours	Able to walk longer distance
1	Before	Stable	Worsened	Stable	Stable	Stable	Stable	Stable	Worsened	Worsened
	After	Stable	**Improved**	Stable	Stable	Stable	Stable	Stable	**Improved**	**Improved**
2	Before	Worsened	Stable	Worsened	Stable	Stable	Stable	Stable	N.A.	N.A.
	After	**Improved**	Stable	**Improved**	Stable	Stable	Stable	Stable	N.A.	N.A.
3	Before	Worsened	Worsened	Stable	Worsened	Stable	Stable	Stable	N.A.	N.A.
	After	**Improved**	**Improved**	**Improved**	**Improved**	Stable	Stable	Stable	N.A.	N.A.
4	Before	Stable	Worsened	Stable	Stable	Stable	Worsened	Stable	N.A.	N.A.
	After	Stable	**Improved**	Stable	Stable	Stable	**Improved**	**Improved**	N.A.	N.A.
5	Before	Worsened	Stable	Worsened	Worsened	Stable	Worsened	Stable	N.A.	N.A.
	After	**Improved**	**Improved**	**Improved**	**Improved**	Stable	**No change**	Stable	N.A.	N.A.
6	Before	Stable	Stable	Worsened	Worsened	Stable	Stable	Stable	N.A.	N.A.
	After	**Improved**	**Improved**	**No change**	**Improved**	Stable	**Improved**	Stable	N.A.	N.A.
7	Before	Worsened	Worsened	Stable	Stable	Stable	N.A.	N.A.	N.A.	N.A.
	After	**Improved**	**Improved**	Stable	**Improved**	Stable	N.A.	N.A.	N.A.	N.A.
8	Before	Stable	Worse	Stable	Worse	Stable	Stable	Stable	N.A.	N.A.
	After	Stable	**Improved**	Stable	**No change**	Stable	**Improved**	**Improved**	N.A.	N.A.
9	Before	Stable	Worse	Stable	Worse	Stable	N.A.	N.A.	N.A.	N.A.
	After	**Improved**	**Improved**	**Improved**	**No change**	**Acquired**	N.A.	N.A.	N.A.	N.A.

*N.A.* Not applicable.

Significant values are in [bold].

The initial steroid dose was 0.5 mg/kg on an alternate-day basis, and, for the nonresponders, the dose was increased up to 1.0 mg/kg on an alternate-day basis.

Regarding the correlation between the steroid dose and its effects, no statistical analysis could be performed because of the small sample size. In Cases 2 and 5, 0.5 mg/kg alternate-day steroid dose (0.25 mg/kg/day) was administered, and motor function improvement was observed. In addition, according to the reports by Toyono et al.^13^, alternate-day administration at 0.5 mg/kg (0.25 mg/kg/day) had a definite effect. As opposed to the minimum dose demonstrated to be effective against DMD (prednisolone at 0.3 mg/kg/day), even lower doses may contribute to improving motor function in patients with FCMD^9,10,13^. Even when the 0.5 mg/kg alternate-day (0.25 mg/kg/day) regimen was ineffective, increasing the steroid doses showed improvement in motor function. This suggests that steroid therapy is effective even at low doses, and its efficacy may be further enhanced by increasing the dose. In addition to improving motor functions, the previously reported effects of steroid therapy include improvement in swallowing functions, intensification of voice volume, and improvement in the peak expiratory flow rate^13–15^. In the present study, two cases achieved a decrease in salivation, and one case witnessed an intensified voice volume (Table 6). Although decreased salivation suggests improvement in swallowing functions, an intensified voice volume may be related to improvement in vital capacity. Similar to steroid therapy for DMD, steroid therapy for FCMD may also contribute to the improvement of swallowing and pulmonary functions. Moreover, six cases exhibited improved motivation, and two cases attained increased vocabulary, indicating that the central nervous system was also affected, in addition to motor functions (Table 5). Patients with FCMD exhibit dysphagia prior to a decrease in the respiratory functions. Therefore, to prevent aspiration and asphyxiation, they sometimes require gastric fistula formation or tracheoesophageal separation^7^. Improvement in the swallowing functions by steroid therapy will lead to a better quality of life among patients with FCMD.

**Table 6:** Improved functions other than motor function.

Patient no.	Motivation	Salivation	Voice volume	Vocabulary
1	I**ncreased**	N.P.	N.P.	N.P.
2	N.P.	N.P.	N.P.	N.P.
3	**Increased**	**Decreased**	N.P.	N.P.
4	**Increased**	**Decreased**	N.P.	N.P.
5	N.P.	N.P.	N.P.	N.P.
6	N.P.	N.P.	N.P.	N.P.
7	**Increased**	N.P.	**Intensified**	N.P.
8	**Increased**	**Decreased**	N.P.	N.P.
9	**Increased**	N.P.	N.P.	**Increased**

*N.P.* Not particularly.

Significant values are in [bold].

Regarding the start/finish times of steroid therapy, our study focused on the patients whose motor functions started regressing. Toyono et al. administered steroid therapy to patients who were capable of walking and whose motor functions reached a developmental plateau, and reported that their walking and moving speed increased^13^. In our study, we administered steroid therapy to patients with severe-type FCMD whose motor functions had started regressing; consequently, the patients became able to sit unaided and attained improved peak motor performance. This finding suggests that starting steroid therapy before the motor functions reach a developmental plateau and start regressing may improve the peak motor performance. However, in FCMD, the timing of the establishment of a developmental plateau of motor functions differs greatly from patient to patient. In severe-type FCMD, patients reach a plateau at around 2 years of age. Hence, steroid therapy should be initiated from an earlier age. In such cases, several issues should be considered, such as preventing adverse reactions, including growth suppression, and inoculating live vaccines. Even during steroid treatment, live vaccines are not always contraindicated, depending on the dose. However, there is little evidence of a clear quantitative relationship between the dose and immunosuppression. The risks and benefits of live vaccines, even at low doses of steroids, must be considered.

Regarding the end time of steroid therapy, the consequences of its long-term administration, in addition to the functional improvement, should be considered, as in steroid therapy for DMD. Once the therapy is found to no longer improve motor functions, it is essential to factor in the risk–benefit balance and decide whether to continue the therapy in consultation with the patients and their guardians. The repercussions of long-term steroid administration also need to be assessed in the future.

The limitations of this study was small sample size, and included the different steroid doses administered to the patients and the rather long time that was needed to confirm the drug compliance. All patients refused to take prednisolone orally because of its bitter taste. Because patients with FCMD have moderate-to-severe mental retardation, their parents often encounter this type of problem during the administration of the medication. In future studies, it will be necessary to increase the number of patients and establish a more robust steroid administration schedule.

The adverse reactions to steroid therapy that occurred during the short period of this study were not sufficiently severe to justify the discontinuation of the therapy; however, one patient did not receive the maximal dose due to parental anxiety regarding the irritability of the patient. Hence, we believe that steroid therapy can be administered safely. In future studies, we need to assess the effects of the long-term administration of these drugs, especially when therapy is initiated at an early age.

As mentioned above, for steroid therapy in FCMD, there are still points of consideration, including the dosage and start/finish times. Nonetheless, the results of this study suggest that steroid therapy is effective in improving/maintaining the motor functions of patients with FCMD for a short period. The fact that steroid therapy improves and maintains the motor functions of patients signifies that it could also improve their quality of life.

## Methods

### Clinical study design

This study was open-label, single-arm clinical study. The protocol was approved by the ethics committee of Tokyo Women’s Medical University (Approval No. 160104, Supplemental Protocol 1, 2) on January 9, 2016 and registered on the Information Network Clinical Trial Registry (UMIN-CTR Unique ID: UMIN000020715) on February 1, 2016. This study was conducted in accordance with the Declaration of Helsinki and was carried out at Tokyo Women’s Medical University Hospital from February 2016 to March 2019. Patients who were being followed at the Department of Pediatrics of Tokyo Women’s Medical University from 2016 to 2019 and who satisfied all of the following requirements were included in the study. (1) Patients genetically diagnosed with FCMD, (2) patients aged 3–10 years whose decline in motor function was clinically ascertained, and (3) consented to undergo steroid therapy provided by the patients or their parents. Verbal and written explanations of the study objectives, methodology, and expected benefits were provided to all the participants and their guardians, and written informed consent was obtained from the guardians of all the children to participate in the study (Supplemental Protocol 1, Sects. 6, 7–9).

### Evaluation methods

Oral steroid therapy was administered to all patients fulfilling the inclusion and exclusion criteria. Their motor functions were evaluated and compared using the GMFM, before and after the onset of steroid therapy.

### Clinical classification of FCMD

The clinical classification of patients with FCMD was based on maximum motor development. Those who could sit on their own or do bottom shuffling were classified as having the typical form of FCMD; those who could walk were classified as having the mild form; and those lacking head control were classified as having the severe form^7^.

### Steroid therapy

Prednisolone was orally administered to the patients at 0.5 mg/kg on an alternate-day basis. In case this therapy proved to be ineffective after 1 month, the steroid dose was increased by 0.2–0.25 mg/kg alternate-day every month up to 1.0 mg/kg alternate-day.

### GMFM

Although the GMFM is a scale that is used for evaluating the motor functions of children with cerebral palsy, it has also been reported to be useful for assessing the motor functions of patients with FCMD^19,20^. The GMFM scale consists of the following 88 items in 5 dimensions: dimension A: lying and rolling (17 items, 51 points); dimension B: sitting (20 items, 60 points); dimension C: crawling and kneeling (14 items, 42 points); dimension D: standing (15 items, 39 points); and dimension E: walking, running, and jumping (24 items, 72 points). Each item was evaluated on a 4-point scale: 0 points: not possible at all; 1 point: barely possible (below 10% of the standard); 2 points: partially possible (≥ 10% and < 100% of the standard); and 3 points: completely possible. For each area, a percent score (actual score/(item count × 3) × 100) was calculated, and the total score is obtained by calculating the average of the five areas. The motor functions of the patients were evaluated using the GMFM and compared before and after the onset of therapy. The evaluation after the therapy was performed 6 months after the drug compliance was confirmed.

### Statistical analysis

For statistical analysis, a Wilcoxon signed-rank test was performed using the SPSS27 software (significance level, *P* ≤ 0.05).

## Data Availability

The datasets generated during and/or analyzed during the current study are available from the corresponding authors on reasonable request.
