# Author Correction: Efficacy of steroid therapy for Fukuyama congenital muscular dystrophy

**DOI:** 10.1038/s41598-022-17588-z

**Published:** 2022-08-02

**Authors:** Terumi Murakami, Takatoshi Sato, Michiru Adachi, Kumiko Ishiguro, Minobu Shichiji, Hisateru Tachimori, Satoru Nagata, Keiko Ishigaki

**Affiliations:** 1 Department of Pediatrics, Tokyo Women’s Medical University School of Medicine, 8‑1 Kawada‑cho, Shinjuku, Tokyo 162‑8666 Japan; 2 Department of Clinical Research, National Hospital Organization Higashisaitama National Hospital, 4147 Kurohama, Hasuda, Saitama 349‑0196 Japan; 3 Department of Rehabilitation, Tokyo Women’s Medical University, 8‑1 Kawada‑cho, Shinjuku, Tokyo 162‑8666 Japan; 4 Department of Clinical Epidemiology, Translational Medical Center, National Center of Neurology and Psychiatry, 4‑1‑1 Ogawa‑Higashi, Kodaira, Tokyo 187‑8551 Japan

Correction to: *Scientific Reports* 10.1038/s41598-021-03781-z, published online 20 December 2021

The Acknowledgements section in the original version of this Article was incomplete.

“This study was funded by a psychological/neurological disease research grant (Grant number: 2-4) provided by the National Center of Neurology and Psychiatry, Japan. We are indebted to Mr. Ben Cooper for his medical writing services.”

now reads:

“This study was funded by a psychological/neurological disease research grant (Grant number: 2-4) provided by the National Center of Neurology and Psychiatry, Japan and supported by SHISEIKAI Scientific Award. We are indebted to Mr. Ben Cooper for his medical writing services.”

The original Article has been corrected.

